# Remediation Strategies for Emergency Medicine Patient Care Milestones

**DOI:** 10.7759/cureus.3557

**Published:** 2018-11-07

**Authors:** Tiffany Murano, Jessica L Smith, Moshe Weizberg

**Affiliations:** 1 Emergency Medicine, Rutgers New Jersey Medical School, Newark, USA; 2 Emergency Medicine, Alpert Medical School of Brown University, Providence, USA; 3 Emergency Medicine, Staten Island University Hospital, Northwell Health, Staten Island, USA

**Keywords:** emergency medicine, remediation, patient care, milestones

## Abstract

Early identification and successful remediation of unachieved emergency medicine (EM) milestones are challenging for program directors. Residents who fail to achieve milestones in the expected time frame will have varied educational needs to course correct, dependent on the year of training, as well as the specific deficiencies to resolve. Experts from the Council of Residency Directors in Emergency Medicine (CORD-EM) Remediation Task Force (RTF) collaborated with the objective to create tools for identifying and remediating residents with deficiencies in patient care milestones (PCMs).

## Introduction

As described by the Accreditation Council for Graduate Medical Education (ACGME), “Milestones are descriptors and targets for resident performance as a resident moves from entry into residency through graduation” [1-2]. As milestones are incorporated into resident assessment, some trainees may not achieve specific milestone levels in the expected time frame. These residents will require remediation to help them achieve training goals. Educators need tools to effectively identify these trainees so that any deficiencies may be addressed as soon as possible. Yet, there are very few assessment tools for emergency medicine (EM) milestones that have been previously validated, leaving residency leadership searching for appropriate tools and strategies. Once a resident is identified, a remediation plan must be developed and implemented; however, the best practices to remediate a deficiency are complex. Best practices differ based on the resident's year of training as well as the actual deficiency. Therefore, the remediation plans that are developed must be tailored to the individual needs of the resident based on the assessment of the program leadership and the Clinical Competency Committee (CCC) before they can be implemented.

Consider clinical scenarios that faculty might encounter: Resident X is a second-year emergency medicine (EM) resident in a three-year training program working in the Emergency Department (ED) in July. He presents a narrow differential diagnosis (DDx) without an appropriate treatment plan and reports that the patient may be discharged. When the faculty member evaluates the patient, he uncovers major discrepancies in the history and key physical examination (PE) findings that necessitate further evaluation and hospital admission. During the program’s CCC meeting, other faculty note similar and consistent deficiencies in the resident’s patient care. The resident falls short in several expected levels of the patient care milestones (PCMs), leaving the CCC wondering about effective remediation strategies to implement at this resident’s stage of training, as well as opportunities for earlier identification of deficiencies during training.  

A second-year EM resident (Resident Y) has just evaluated a patient with a history of coronary artery disease, hypertension, and myocardial infarction. The patient presented to the ED with chest pain, marked hypertension, and new T-wave inversions. The nurse asks why the patient has been admitted to an unmonitored bed. The resident says it is because “his vital signs were stable.” Later that day, this resident tells you that he wants to discharge a patient with new-onset diabetes. However, the patient has no health insurance, no means of obtaining medication, and no follow-up physician.

Although Hauer et al. described a general approach to the remediation of physician performance deficits, it can be difficult to translate the deficient skill into a specific behavior to be targeted in a remediation plan [3]. With the adoption of the milestones, new remediation tools are required to address milestone-based deficiencies and to craft milestone-based remediation plans. 

The objective of this project was to determine the best practices for remediation and create tools for identification, assessment, and strategies for remediation of deficiencies in patient care milestones.  

## Materials and methods

The Council of Residency Directors in Emergency Medicine-Remediation Task Force (CORD-EM RTF) is comprised of residency leadership from EM training programs all across the United States. The CORD-EM RTF was divided into four working subgroups based on the six core-competencies: (1) patient care, (2) medical knowledge, (3) professionalism and interpersonal communication skills, and (4) practice-based learning and improvement and systems-based practice. Each of the subgroups was tasked with focusing on the stated objective: determination of best practices for remediation for each of the given competencies and creation of a toolkit that program directors can utilize to identify and remediate residents.

The authors’ group, comprised of EM residency program leadership with over 60 years of collective graduate medical education (GME) experience, focused on the PCMs. The PCM subgroup had telephone and email correspondence, as well as face-to-face meetings twice per year over a two-year period, to discuss the objectives and collaborate.

The first step was a literature search of the best practices for remediation of patient care (PC). PubMed and MEDLINE databases were used to search for literature pertaining to remediation of patient care milestones. For the PubMed database search, the following medical subject heading (MeSH) terms were used:  "Education, Medical, Graduate" OR "Internship and Residency," OR "Clinical Competence," AND "Emergency Medicine," AND "Curriculum," AND "last 10 years." The following additional MeSH terms were used to search for literature pertaining to specific PCMs:  "Resuscitation," "Diagnostic Imaging," "Diagnosis," "Physiologic Phenomena," "Airway Management," "Therapeutics," "Wounds and Injuries," and "Catheters." Similar terms were used in the MEDLINE data search. Only articles in the English language were considered for review. In addition, only articles pertaining to methods of improving patient care skills in postgraduate physician learners were considered for review and utilization. Since there is significant overlap in many aspects of patient care across all specialties, the articles were not limited to those pertaining to only emergency medicine. The search yielded 38 articles in total and the articles were screened for content that was focused on curricula for EM procedures, assessment tools, and resources used for the education of PCMs. A total of 17 articles were selected for review from the literature search; 14 of these articles were utilized in the creation of the PCM rubric. These 14 articles were divided into the following categories: simulation/task trainers (8), curriculum (2), assessment tools (3), and free open access medical education (1).

Next, the group worked to create a tool that would assist in the identification of residents in need of remediation of PCMs. The EM milestones list the standardized direct observation tool (SDOT) to assess milestone achievement. However, the previously developed emergency medicine SDOT is not milestone-based and may be difficult to translate when performing milestone evaluations. Therefore, the Patient Care Milestone Standardized Direct Observation Tools (PC-mSDOTs) was created to reflect the influence of the new milestones (m) on the SDOT.

Seven mSDOTs were developed for each EM training year as depicted in Table 1. The EM-3 and EM-4 years were combined into one mSDOT for use in either three- or four-year programs, as residents in their final year of training are expected to perform at the higher milestone levels. The evaluator is expected to indicate whether the level has been achieved, needs improvement, or was not observed. Faculty comments and review of the mSDOT with the resident in real time is expected. Residents may also provide comments.  

**Table 1:** The Seven Patient Care Milestone Standardized Direct Observation Tools (PC-mSDOTs) Each PC-mSDOT evaluates a specific set of milestones as indicated below. Each EM-training year has a set of seven PC-mSDOTs, which have varying milestone levels depending on the EM-training year of the resident. EM: emergency medicine; PC: patient care; PCM: patient care milestone

PCM in each of the Standardized Direct Observation Tools (PC-mSDOTs) for each EM-training year: 1. PC 1-4 2. PC 5-8 3. PC 9, 10 (Airway) 4. PC 9, 11 (Procedural Sedation) 5. PC 9, 11, 13 (Anesthesia/Wound Management) 6. PC 9, 12 (Ultrasound) 7. PC 9, 14 (Vascular Access)	
EM-Training Year	Milestone Level Descriptors in mSDOTs
EM-1	1 and 2
EM-2	2 and 3
EM-3 and EM-4	3 and 4

The final step was the development of the PCM rubric. The ACGME EM Milestones were used as a guide to approaching remediation of each PCM at each level. Using the results of the literature search, as well as the combined program director experience with successful remediation practices from the subgroup, recommendations for potential remediation strategies were collated into the rubric. Only proficiency levels 1-4 were targeted, as level 5 represents a post-residency aspirational achievement. 

## Results

Assessment and remediation of patient care milestones

The creation of the PC-mSDOT in conjunction with the remediation rubric provides a new resource for the early identification of residents in need of remediation, as well as strategies for the development and the implementation of a plan based on the CORD-EM RTF's best practices and expert consensus.

*Early Identification: A New Assessment Tool *– *The PC-mSDOT*

The assessment of resident performance can occur in or out of the clinical setting and can utilize various assessment methods, the combination of which provides different degrees of standardization [4]. Direct observation can provide valuable information regarding a resident’s performance of PC. The SDOT was developed to obtain partial standardization via a structured observer assessment in the clinical setting and has been shown to have good inter-rater reliability [5-6]. By incorporating the PCMs into the SDOT and thus creating the PC-mSDOT, it is our hope that residency leadership may have a tool that will more readily indicate when a resident has deficiencies in these areas. The evaluator is expected to indicate whether the level has been achieved, needs improvement, or was not observed. Faculty comments and review of the mSDOT with the resident in real time is expected. Residents may also provide comments.  

**Figure 1:**
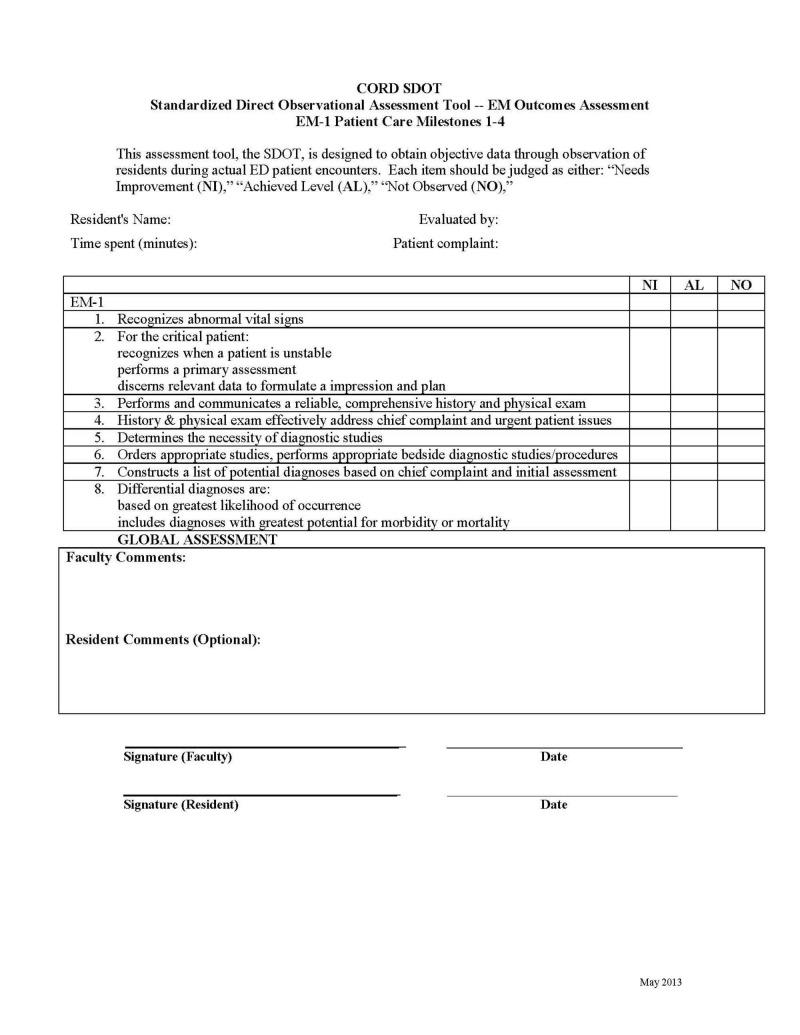
Example of one of the CORD PC-mSDOTs. This PC-mSDOT is to be used to assess first year emergency medicine residents' performance in patient care milestones 1-4. CORD PC-mSDOTs: Council of Residency Directors in Emergency Medicine Patient Care Milestone Standardized Direct Observation Tools; ED: emergency department; EM: emergency medicine

An example of the PC-mSDOT is depicted in Figure 1. The full complement of PC-mSDOTs have been posted on the Council of Residency Directors (CORD) website for use by all EM residency programs and may be accessed through the following link: http://www.cordem.org/resources/residency-management/cord-standardized-assessment-methods/

Formulating a Remediation Plan: Consensus Recommendations

Each resident has distinct strengths and weaknesses and some may struggle to progress to expected milestone levels at different points during training. Recognizing that there is no “one-size-fits-all” remediation curriculum, plans that are unique to the individual resident’s deficiencies are needed. The literature search supported the use of simulation and task trainers in the education and evaluation of many of the PCMs [7-14]. Three articles had assessment tools for procedure-based PCMs [15-17]. There were two articles that were curriculum-based and one article that supported the use of free open access medical education as a means of increasing knowledge base for emergency procedures [18-20]. The Patient Care Remediation Task Force (PC-RTF) created a compilation of consensus remediation practices utilizing documented methodology from our literature search and personal experience that is linked to levels 1 - 4 of 14 (Table 2). 

**Table 2:** Patient Care Remediation Rubric. Emergency Medicine Patient Care Milestones and Suggested Remediation Strategies by Proficiency Level (with General Suggested Evaluation Methods from the ACGME/ABEMa Milestones Project) Emergency medicine patient care milestones and suggested remediation strategies by proficiency level (with general suggested evaluation methods from the ACGME/ABEMa Milestones Project) [1]. ABEM: American Board of Emergency Medicine; ACGME: Accreditation Council for Graduate Medical Education; BVM: bag valve mask; CHADS score: Congestive Heart Failure, Hypertension, Age, Diabetes Mellitus, and Stroke/Transient Ischemia Attack History; DNR: do not resuscitate; ECMO: extracorporeal membrane oxygenation; ED: emergency department; eFAST: extended focused assessment with sonography in trauma; hr: hour; ICU: intensive care unit; Nexus: National Emergency X-Radiography Utilization Study; OSCE: objective structure clinical exam; PC-mSDOT - Patient Care Milestone Standardized Direct Observation Tools; PC: patient care; PCP: primary care physician; PERC: pulmonary embolism rule-out criteria; prn: as needed; PT/OT: physical therapy/occupational therapy; RSI: rapid sequence intubation; US: ultrasound The copyright owners grant third parties the right to use the Emergency Medicine Milestones on a non-exclusive basis for educational purposes.

LEVEL 1	LEVEL 2	LEVEL 3	LEVEL 4
PC1: Emergency Stabilization: Prioritizes critical initial stabilization action and mobilizes hospital support services in the resuscitation of a critically ill or injured patient and reassesses after stabilizing intervention.			
Recognizes abnormal vital signs.	Recognizes when a patient is unstable requiring immediate intervention.	Manages and prioritizes critically ill or injured patients.	Recognizes in a timely fashion when further clinical intervention is futile.
		Prioritizes critical initial stabilization actions in the resuscitation of a critically ill or injured patient.	
	Performs a primary assessment on a critically ill or injured patient.	Reassesses after implementing a stabilizing intervention	Integrates hospital support services into a management strategy for a problematic stabilization situation.
	Discerns relevant data to formulate a diagnostic impression and plan.	Evaluates the validity of a DNR order.	
PC1 SUGGESTED REMEDIATION METHODS: SDOT, observed resuscitations, simulation, checklist, videotape review			
Have the resident spend extra time in the simulation lab with cases that have abnormal vital signs.	Have the resident do bedside presentations to give faculty an opportunity to highlight signs of instability.	Have the resident review the triage information and articulate any concerns before seeing the patient.	Have the resident review the literature and present a lecture on validated criteria for terminating a resuscitation.
Require the resident to specifically address the vital signs on every case presentation.	Have the resident review the triage information before seeing a patient.	Promote early presentations via the technique: “You have X time to see the patient and then find me to present.”	Have the resident spend extra time in the simulation lab with cases that involve problematic stabilization situations (i.e., a contaminated patient, a patient requiring ECMO, etc.) requiring hospital support services.
	Have the resident spend extra time in the simulation lab with cases that have chief complaints that could indicate critical conditions and review elements of the history and physical that suggest critical conditions.	Have the resident practice team leadership mock simulations.	
	Engage in oral board style case review.	Ask the resident to actively engage the attending as needed during a resuscitation.	
		Encourage the resident to engage the nurses frequently during a resuscitation to get support, ideas, and collaboration.	
PC2: Performance of Focused History and Physical Exam: Abstracts current findings in a patient with multiple chronic medical problems and, when appropriate, compares with a prior medical record and identifies significant differences between the current presentation and past presentations.			
Performs and communicates a reliable, comprehensive history and physical exam.	Performs and communicates a focused history and physical exam which effectively addresses the chief complaint and urgent patient issues.	Prioritizes essential components of a history given a limited or dynamic circumstance.	Synthesizes essential data necessary for the correct management of patients using all potential sources of data.
		Prioritizes essential components of a physical examination given a limited or dynamic circumstance.	
PC2 SUGGESTED REMEDIATION METHODS: Global ratings of live performance, checklist assessments of live performance, SDOT, oral boards, simulation			
Have the resident work one-on-one with an attending who will review every history and physical exam. The attending will provide feedback on obtaining the pertinent historical information and performing a focused physical exam.	Adjust resident pace: have the resident get one patient “right” before picking up another.	Require extra time in the simulation lab with cases involving difficult histories and complex physical exams.	Require the resident to pursue all sources of data on every patient (i.e., present the findings from the old chart, call the nursing home to find out what happened to the patient).
	Require bedside presentations.		
	Ask the resident to review the triage information before seeing the patient to prompt the focus of the encounter.	Engage in oral board style case review.	Require extra time in the simulation lab with cases requiring multiple sources of data to obtain a sufficient history.
Have the resident shadow “master clinicians” or more senior residents (i.e., Chiefs) for a successful frame of reference.	Engage in simulated patient encounters involving urgent patient issues, give immediate feedback and monitor progress at regular intervals.		
	Engage in oral board style case review.	Encourage direct observation and evaluation by faculty members.	Engage in oral board style case review.
	Encourage direct observation and evaluation by faculty members.		Encourage direct observation and evaluation by faculty members.
PC3: Diagnostic Studies: Applies the results of diagnostic testing based on the probability of disease and the likelihood of test results altering management.			
Determines the necessity of diagnostic studies.	Orders appropriate diagnostic studies.	Prioritizes essential testing.	Uses diagnostic testing based on the pre-test probability of disease and the likelihood of test results altering management.
	Performs appropriate bedside diagnostic studies and procedures.	Interprets results of a diagnostic study, recognizing limitations and risks, seeking interpretive assistance when appropriate.	Practices cost-effective ordering of diagnostic studies.
		Reviews risks, benefits, contraindications, and alternatives to a diagnostic study or procedure.	Understands the implications of false positives and negatives for post-test probability.
PC3 SUGGESTED REMEDIATION METHODS: SDOT, oral boards, standardized exams, chart review, simulation			
Require the resident to review validated decision rules for diagnostic tests (i.e., PERC, Nexus, CHADS, Ottawa).	Require bedside presentations of findings to assist in developing diagnostic and therapeutic workup plans.	Have the resident engage in simulated patient encounters involving decisions about testing, give immediate feedback and monitor progress at regular intervals.	Have the resident spend extra time in the simulation lab with cases requiring the resident to determine a pre-test probability and utility of diagnostic testing (i.e., low-risk chest pain, suspected pulmonary embolism).
	Encourage the use of validated diagnostic algorithms to support management plans (i.e., PERC, Nexus, CHADS, Ottawa).	Engage in oral board style case review.	
	Closely monitor in-training exam scores and other residency assignments, as a deficiency in PC may be a sign of a larger knowledge base deficit.	Encourage direct observation and evaluation by faculty members.	
		Closely monitor in-training exam scores and other residency assignments, as a deficiency in PC may be a sign of a larger knowledge base deficit.	
PC4: Diagnosis: Based on all of the available data, narrows and prioritizes the list of weighted differential diagnoses to determine appropriate management.			
Constructs a list of potential diagnoses based on chief complaint and initial assessment.	Constructs a list of potential diagnoses, based on the greatest likelihood of occurrence.	Uses all available medical information to develop a list of ranked differential diagnoses including those with the greatest potential for morbidity or mortality.	Synthesizes all of the available data and narrows and prioritizes the list of weighted differential diagnoses to determine appropriate management.
	Constructs a list of potential diagnoses with the greatest potential for morbidity or mortality.	Correctly identifies “sick versus not sick” patients.	
		Revises a differential diagnosis in response to changes in a patient’s course over time.	
PC4 SUGGESTED REMEDIATION METHODS: SDOT as baseline, global ratings, simulation, oral boards, chart review			
Require the resident to develop five differential diagnoses for each patient encounter, based on the greatest likelihood of occurrence.	Have the resident and supervising faculty include clinical reasoning for the differential during the oral case presentation.	Have the resident and supervising faculty include clinical reasoning for the differential during the oral case presentation and again at the wrap-up of the case.	Require the resident to determine the two most likely differential diagnoses for every patient.
Encourage the use of mnemonics for differential diagnoses (i.e., VINDICATE)	Have the resident engage in simulated patient encounters, give immediate feedback and monitor progress at regular intervals.	Have the resident engage in simulated patient encounters, give immediate feedback and monitor progress at regular intervals.	
Have the resident include clinical reasoning for the differential diagnosis during the oral case presentation.	Engage in oral board style case review.	Require the resident to spend additional clinical time managing critical care patients.	
Utilize direct observation and assessment by core faculty members.	Ask the resident to prioritize the differential diagnosis to identify the most likely and deadliest diagnoses in order to guide work-up.	Engage in oral board style case review.	Utilize direct observation and assessment by core faculty members.
	Utilize direct observation and assessment by core faculty members.	Utilize direct observation and assessment by core faculty members.	
	Closely monitor in-training exam scores and other residency assignments as a deficiency in differential diagnosis is often a sign of a knowledge deficit.	Closely monitor in-training exam scores and other residency assignments as a deficiency in differential diagnosis is often a sign of a knowledge deficit.	
PC5: Pharmacotherapy: Selects and prescribes, appropriate pharmaceutical agents based upon relevant considerations, such as mechanism of action, intended effect, financial considerations, possible adverse effects, patient preferences, allergies, potential drug-food and drug-drug interactions, institutional policies, and clinical guidelines; and effectively combines agents and monitors and intervenes in the advent of adverse effects in the ED.			
Knows the different classifications of pharmacologic agents and their mechanism of action.	Applies medical knowledge for selection of an appropriate agent for therapeutic intervention.	Considers array of drug therapy for treatment. Selects appropriate agent based on the mechanism of action, intended effect, and anticipates potential adverse side effects.	Selects the appropriate agent based on the mechanism of action, intended effect, possible adverse effects, patient preferences, allergies, potential drug-food and drug-drug interactions, financial considerations, institutional policies, and clinical guidelines, including patient’s age, weight, and other modifying factors.
Consistently asks patients for drug allergies.	Considers potential adverse effects of pharmacotherapy.	Considers and recognizes potential drug to drug interactions.	
PC5 SUGGESTED REMEDIATION METHODS: SDOT, portfolio, simulation, oral boards, global ratings, medical knowledge examinations			
Require the resident to read about various pharmacologic agents and present the information from their reading to an attending (i.e., “Read about beta blockers and come teach me about it tomorrow.”).	Require the resident to include the patient’s drug allergies in his presentations.	Assign the resident to participate in patient safety initiatives that deal with medication errors.	Require the resident to document a search for drug-drug interactions on every patient.
Require the resident to explain his/her medication choices when presenting a plan.	Require the resident to participate in simulation cases in which he must specify the names and dosages of medications.		Have the resident spend extra time in the simulation lab with cases involving complex medication choices.
Require the resident to document drug allergies on every patient encounter.	Require the resident to identify risks and benefits of any medications he/she recommends being administered.	Have the resident document a search for drug-drug interactions in 10 patient encounters.	
	Provide the resident with a list of commonly prescribed medications (e.g., antibiotics and antihypertensive agents) and have them research/report costs. Engage in oral board style case review.		
	Encourage direct observation and evaluation by faculty members.	Require the resident to document a search for drug-drug interactions on every patient.	
	Closely monitor in-training exam scores and other residency assignments as deficits in PC5 may be a sign of a knowledge base deficit.	Engage in oral board style case review.	
		Encourage direct observation and evaluation by faculty members.	
PC6: Observation and Reassessment: Re-evaluates patients undergoing ED observation (and monitoring) and using appropriate data and resources, determines the differential diagnosis, treatment plan, and disposition.			
Recognizes the need for patient reevaluation.	Monitors that necessary therapeutic interventions are performed during a patient’s ED stay.	Identifies which patients will require observation in the ED.	Considers additional diagnoses and therapies for a patient who is under observation and changes treatment plan accordingly.
		Evaluates effectiveness of therapies and treatments provided during observation.	Identifies and complies with federal and other regulatory requirements, including billing, which must be met for a patient who is under observation.
		Monitors a patient’s clinical status at timely intervals during their stay in the ED.	
PC6 SUGGESTED REMEDIATION METHODS: SDOT, multi-source feedback, oral boards, simulation			
Require the resident to reevaluate every patient at specific intervals.	Require the resident to “check-in” with the attending at frequent intervals to report the progress of therapeutic interventions.	In disposition planning, require the resident to justify whether observation is indicated for commonly encountered conditions (e.g., asthma exacerbation, allergic reaction, abdominal pain of unclear etiology, etc.).	Require the resident to complete a rotation in an observation unit.
Require the resident to update the attending about the patient's progress at specific intervals.	Require the resident to describe the reevaluation and discharge plan for all patients prior to discharge.	Review resident documentation of patient reevaluation including time, assessment, and interventions.	Require the resident to research billing requirements for observation patients and present the findings to the group in an educational setting.
	Require the resident to complete oral boards or simulation cases that require the reassessment of interventions.	Engage in oral board style case review.	
	Have the resident engage in simulated patient encounters, give immediate feedback, and monitor progress at regular intervals.	Encourage direct observation and evaluation by faculty members.	
	Ask the resident to do frequent rounds on patients to ensure critical interventions are done in a timely fashion: include the ABC’s, assessment of patient discomfort, urgent consultations, etc.	Have the resident do clinical shifts in an observation unit.	
PC7: Disposition: Establishes and implements a comprehensive disposition plan that uses appropriate consultation resources; patient education regarding diagnosis; treatment plan; medications; and time and location-specific disposition instructions.			
Describes basic resources available for care of the emergency department patient.	Formulates a specific follow-up plan for common ED complaints with appropriate resource utilization.	Formulates and provides patient education regarding diagnosis, treatment plan, medication review and PCP/consultant appointments for complicated patients. Involves appropriate resources (e.g., PCP, consultants, social work, PT/OT, financial aid, care coordinators) in a timely manner. Makes correct decision regarding admission or discharge of patients. Correctly assigns admitted patients to an appropriate level of care (ICU/ Telemetry/ Floor/ Observation Unit).	Formulates sufficient admission plans or discharge instructions, including future diagnostic/therapeutic interventions for ED patients. Engages patient or surrogate to effectively implement a discharge plan.
PC7 SUGGESTED REMEDIATION METHODS: SDOT, shift evaluations, simulation cases / Objective Structure Clinical Exam (OSCE), multi-source feedback, chart review			
Review with the resident the available resources in the department.	Require the resident to personally discharge 10 patients and review medications, follow-up information, and return precautions while being directly observed.	Have the resident personally make follow-up appointments for some patients to evaluate the accessibility and timeliness of primary or specialty care.	Require the resident to review all discharge instructions with the attending prior to disposition.
	Require the resident to describe the discharge plan, including patient or family concerns, safety issues, financial barriers, or reliability of compliance prior to discharging patients from the ED.	Require the resident to complete oral board cases that provide a range of acuity levels for disposition.	Engage in oral board review cases that involve communicating with a surrogate.
		Require the resident to discharge standardized patients with a variety of issues while being observed.	
PC8: Multi-tasking (Task-switching): Employs task switching in an efficient and timely manner in order to manage the ED.			
Manages a single patient amidst distractions.	Task switches between different patients.	Employs task switching in an efficient and timely manner in order to manage multiple patients.	Employs task switching in an efficient and timely manner in order to manage the ED.
PC8 SUGGESTED REMEDIATION METHODS: Simulation, SDOT, mock oral examination, multi-source feedback			
Require the resident to focus on one patient at a time. Resident must complete the history and physical, present the patient, enter orders, and ensure the plan is complete, prior to picking up the next patient.	Ask that patients are presented shortly after assessment and actions are prioritized together.	Establish expectations for the resident based on year of training – e.g., interns focus on assessment of the ABC’s vs. a senior who should step back to run a resuscitation and delegate, rather than place a central line for example. Expectations should be clarified prior to the arrival of the patient(s).	Provide direct guidance from attendings who are particularly good at “moving the Department.”
	Emphasize the need to delegate non-physician tasks to nurses/techs when there are physician-only responsibilities waiting.	Have the resident run multiple patient scenarios in the simulation lab.	
	Have the resident engage in simulated encounters of multiple patients, give immediate feedback and monitor progress at regular intervals.	Engage in oral board style case review with multiple patient encounters.	Give the resident the opportunity to function as a “pre-attending,” requiring them to manage the entire ED.
Utilize direct observation and redirect the resident as needed to prioritize correctly.	Engage in oral board style case review with multiple patient encounters.	Utilize direct observation and redirect the resident as needed to prioritize correctly.	
	Encourage direct observation and evaluation by faculty members.	Obtain and review productivity data (i.e., patients/hr) and compare this to peers or local/national expectations.	Utilize direct observation and redirect the resident as needed to prioritize correctly.
		Set a specific expectation with the resident that they will see “X” patients per hour or per shift.	
PC9: General Approach to Procedures: Performs the indicated procedure on all appropriate patients (including those who are uncooperative, at the extremes of age, hemodynamically unstable, and those who have multiple co-morbidities, poorly defined anatomy, high risk for pain or procedural complications, sedation requirement), takes steps to avoid potential complications, and recognizes the outcome and/or complications resulting from the procedure.			
Identifies pertinent anatomy and physiology for a specific procedure. Uses appropriate universal precautions.	Performs patient assessment, obtains informed consent and ensures monitoring equipment is in place in accordance with patient safety standards.	Determines a backup strategy if initial attempts to perform a procedure are unsuccessful.	Performs indicated procedures on any patients with challenging features (e.g., poorly identifiable landmarks, at extremes of age, or with co-morbid conditions).
	Knows indications, contraindications, anatomic landmarks, equipment, anesthetic, and procedural technique, and potential complications for common ED procedures.	Correctly interprets the results of a diagnostic procedure.	Performs the indicated procedure, takes steps to avoid potential complications, and recognizes the outcome and/or complications resulting from the procedure.
	Performs the indicated common procedure on a patient with moderate urgency who has identifiable landmarks and a low/moderate risk for complications.		
	Performs post-procedural assessment and identifies any potential complications.		
PC9 SUGGESTED REMEDIATION METHODS: Procedural competency forms, checklist assessment of procedure and simulation lab performance, global ratings			
Require the resident to review the relevant anatomy and physiology for the procedure in a procedure text or video.	Have the resident spend extra time in the simulation lab or cadaver lab to review the relevant procedures.	Have the resident spend extra time in the simulation lab or cadaver lab to review the relevant procedures.	Have the resident spend extra time in the cadaver lab or simulation lab to practice the specific procedure under direct guidance.
The resident must then present the information to the attending prior to performing the procedure.	Have the resident prepare a summary of common procedures, indications, contraindications, anatomic landmarks, equipment, anesthetic, and procedural technique, and potential complications for common ED procedures to review with a mentor.	Have the resident prepare and present a summary of the backup strategies to employ for unsuccessful common ED procedures.	
		Have the resident review and present the interpretation of results of common diagnostic procedures.	
PC10: Airway Management: Performs airway management on all appropriate patients (including those who are uncooperative, at the extremes of age, hemodynamically unstable, and those who have multiple co-morbidities, poorly defined anatomy, high risk for pain or procedural complications, sedation requirement), takes steps to avoid potential complications, and recognize the outcome and/or complications resulting from the procedure.			
Describes upper airway anatomy.	Describes elements of airway assessment and indications impacting the airway management.	Uses airway algorithms in decision making for complicated patients employing airway adjuncts as indicated.	Performs airway management in any circumstance taking steps to avoid potential complications, and recognizes the outcome and/or complications resulting from the procedure.
	Describes the pharmacology of agents used for rapid sequence intubation, including specific indications and contraindications.	Performs rapid sequence intubation in patients using airway adjuncts.	Performs a minimum of 35 intubations.
Performs basic airway maneuvers or adjuncts (jaw thrust/chin lift/oral airway/nasopharyngeal airway) and ventilates/oxygenates patient using BVM.	Performs rapid sequence intubation in patients without adjuncts.	Implements post-intubation management.	Demonstrates the ability to perform a cricothyrotomy.
	Confirms proper endotracheal tube placement using multiple modalities.	Employs appropriate methods of mechanical ventilation based on specific patient physiology.	Uses advanced airway modalities in complicated patients.
PC10 SUGGESTED REMEDIATION METHODS: Airway Management Competency Assessment Tool (CORD), Airway Management Assessment Cards, SDOT checklist, procedure log, simulation			
Require review of airway procedure videos. Have the resident spend extra time in the simulation lab or cadaver lab to review the basic airway management techniques.	Have the resident spend extra time in the simulation lab or cadaver lab to review intubation techniques. Require a minimum number of simulated airway management with direct supervision. Require the resident to research the pharmacology of agents used for RSI and present it at the resident conference. Send the resident to an airway management course.	Have the resident spend extra time in the simulation lab or cadaver lab to manage difficult airways and select appropriate ventilator settings. Have the resident prepare and present a summary of post-intubation management. Send the resident to an airway management course.	Have the resident spend extra time in the simulation lab or cadaver lab to manage difficult airways. Have the resident spend extra time in the simulation lab or cadaver lab to perform cricothyrotomy. Send the resident to an airway management course.
PC11: Anesthesia and Acute Pain Management: Provides safe acute pain management, anesthesia, and procedural sedation to patients of all ages regardless of the clinical situation.			
Discusses with the patient indications, contraindications and possible complications of local anesthesia.	Knows the indications, contraindications, potential complications and appropriate doses of analgesic/sedative medications.	Knows the indications, contraindications, potential complications and appropriate doses of medications used for procedural sedation.	Performs procedural sedation providing effective sedation with the least risk of complications and minimal recovery time through selective dosing, route, and choice of medications.
Performs local anesthesia using appropriate doses of local anesthetic and appropriate technique to provide skin to sub-dermal anesthesia for procedures.	Knows the anatomic landmarks, indications, contraindications, potential complications and appropriate doses of local anesthetics used for regional anesthesia.	Performs patient assessment and discusses with the patient the most appropriate analgesic/sedative medication and administers in the most appropriate dose and route.	
		Performs pre-sedation assessment, obtains informed consent and orders appropriate choice and dose of medications for procedural sedation.	
		Obtains informed consent and correctly performs regional anesthesia.	
		Ensures appropriate monitoring of patients during procedural sedation.	
PC11 SUGGESTED REMEDIATION METHODS: Procedural competency forms, checklist assessment of procedure and simulation lab performance, global ratings, patient survey, chart review			
Utilize direct supervision of the resident discussing the administration of local anesthesia with a specified number of patients.	Have the resident review and discuss common analgesic and sedative medications.	Have the resident review and discuss common medications for procedural sedation.	Utilize direct supervision of the resident performing procedural sedation on a specified number of patients.
Have the resident spend extra time in a suture lab.	Have the resident review procedure videos on regional anesthesia.	Observe the resident in the simulation lab practicing informed consent and pre-sedation conversations with mock patients.	
		Utilize direct supervision of the resident performing pre-sedation assessment on a specified number of patients and provide appropriate feedback.	
		Utilize direct supervision of the resident performing procedural sedation on a specified number of patients.	
PC12: Other Diagnostic and Therapeutic Procedures: Goal-directed focused ultrasound (Diagnostic/Procedural): Uses goal-directed focused ultrasound for the bedside diagnostic evaluation of emergency medical conditions and diagnoses, resuscitation of the acutely ill or injured patient, and procedural guidance.			
Describes the indications for emergency ultrasound.	Explains how to optimize ultrasound images and Identifies the proper probe for each of the focused ultrasound applications.	Performs goal-directed focused ultrasound exams.	Performs a minimum of 150 focused ultrasound examinations.
	Performs an eFAST.	Correctly interprets acquired images.	
PC12 SUGGESTED REMEDIATION METHODS: OSCE, SDOT, videotape review, written examination, checklist			
Have the resident review and report on the common indications for emergency ultrasound.	Have the resident review procedural videos on emergency ultrasound.	Require the resident to perform a specified number of ultrasound exams under direct supervision.	Require the resident to perform the required number of studies.
	Require the resident to perform a specified number of eFAST exams under direct supervision.	Require additional time for the resident to participate in ultrasound image review.	Require an US Elective/course.
	Require an US elective.	Require an US elective/course.	
PC13: Other Diagnostic and Therapeutic Procedures: Wound Management: Assesses and appropriately manages wounds in patients of all ages regardless of the clinical situation.			
Prepares a simple wound for suturing (identify appropriate suture material, anesthetize wound, and irrigate).	Uses medical terminology to clearly describe/classify a wound (e.g., stellate, abrasion, avulsion, laceration, deep vs superficial). Educates patients on appropriate outpatient management of their wound.	Performs complex wound repairs (deep sutures, layered repair, corner stitch).	Achieves hemostasis in a bleeding wound using advanced techniques such as cautery, ligation, deep suture, injection, topical hemostatic agents, and tourniquet.
Demonstrates sterile technique.	Classifies burns with respect to depth and body surface area.	Manages a severe burn.	Repairs wounds that are high risk for cosmetic complications (such as eyelid margin, nose, ear).
Places a simple interrupted suture.	Compares and contrasts modes of wound management (adhesives, Steri-strips, hair apposition, staples).	Determines which wounds should not be closed primarily.	Describes the indications for and steps to perform an escharotomy.
	Identifies wounds that require antibiotics or tetanus prophylaxis.	Demonstrates appropriate use of consultants. Identifies wounds that may be high risk and require more extensive evaluation (example: x-ray, ultrasound, and/or exploration).	
PC13 SUGGESTED REMEDIATION METHODS: Direct observation, procedure checklist, medical knowledge quiz, portfolio, global ratings, procedure log			
Have the resident spend extra time in a suture lab.	Require the resident to review a textbook chapter on wound management and describe all wounds accurately in the medical record.	Have the resident spend extra time in a suture lab managing complex lacerations.	Require the resident to review procedure videos on complex wound management.
	Require the resident to review and discuss basic burn assessment with a mentor.	Require the resident to watch procedure videos on complex wound management.	Require the resident to review procedure videos on escharotomy.
	Require the resident to watch procedure videos on wound management.	Require the resident to review and discuss burn management with a mentor.	Consider or require an elective in a burn unit.
	Utilize direct supervision of the resident providing wound management instructions to patients.	Have the resident spend time in the simulation lab managing simulated patients with severe burns.	
		Consider or require an elective in a burn unit.	
PC14: Other Diagnostic and Therapeutic Procedures: Vascular Access: successfully obtains vascular access in patients of all ages regardless of the clinical situation.			
Performs a venipuncture.	Describes the indications, contraindications, anticipated undesirable outcomes, and complications for the various vascular access modalities. Inserts an arterial catheter. Assesses the indications in conjunction with the patient anatomy/pathophysiology and select the optimal site for a central venous catheter. Inserts a central venous catheter using ultrasound and universal precautions. Confirms appropriate placement of central venous catheter. Performs intraosseous access.	Inserts a central venous catheter without ultrasound when appropriate.	Successfully performs 20 central venous lines.
Places a peripheral intravenous line.		Places an ultrasound-guided deep vein catheter (e.g., basilic, brachial, and cephalic veins).	Routinely gains venous access in patients with difficult vascular access.
Performs an arterial puncture.			
PC14 SUGGESTED REMEDIATION METHODS: Knowledge assessment using multiple choice questions, checklist driven task analysis, procedure log			
Have the resident perform the relevant procedures under direct supervision.	Require the resident to review and discuss a textbook chapter on vascular access and describe the indications for each.	Have the resident spend extra time in the simulation lab practicing blind access.	Require the resident to perform extra shifts in critical care areas (in the ED or in ICU’s) to complete the required number of central lines.
	Have the resident review and present chest X-rays of appropriate and inappropriate placement of central lines.		
Practice venipuncture skills on task trainers and vascular mannequins.	Require the resident to perform the relevant procedures under direct supervision.	Require the resident to perform the relevant procedures under direct supervision.	Require the resident to review procedure videos on vascular access in difficult patients.
	Require the resident to review procedure videos on vascular access.	Require the resident to review procedure videos on the relevant procedures.	Require the resident to practice vascular access skills with task trainers and mannequins.
	Require the resident to practice vascular access skills with task trainers and mannequins.	Require the resident to practice vascular access skills with task trainers and mannequins.	

Implementing a Remediation Plan: Combining Toolkit Options 

The PC-mSDOT may be administered to all residents or residents who have been identified as having or potentially having PCM deficiencies. Administering the PC-mSDOT to all residents early in the academic year establishes a baseline, and deficiencies may be identified expeditiously. A second PC-mSDOT may then be administered later in the academic year to track the progress of the remediation. The PC-mSDOT may be used to assess performance in both the clinical and extra-clinical settings.

Using the PCM remediation rubric in Table 2, residency leadership may readily access suggested remediation methods when it is discovered that there are specific deficiencies that require additional resources. There are also suggested assessment methods linked to each PCM that may be used to identify deficiencies and track progress. Below each PCM level, there are remediation strategies and tools which may be incorporated into individualized plans. This may be used for residents with deficiencies at any point in their training.

Referring back to the resident scenarios presented in the Introduction and based on the RTF-PC toolkit, we offer sample remediation plans. 

Resident X has deficiencies in PC2, PC4, and PC7 and does not meet level 1 for these PCMs. Applying the remediation rubric, the PD would refer to level 1 of the three individual PCMs and may develop a remediation plan to include: 

During clinical shifts for the EM block, Resident X will be required to:

▫    Work one-on-one with faculty who will review every history and PE with the resident;

▫    Have direct and immediate feedback from faculty with particular attention on the history and focused PE skills;

▫    Shadow the senior resident on shift while s/he performs a history and PE (to establish a successful frame of reference);

▫    Develop a list of at least five differential diagnoses for each patient encounter based on the likelihood of occurrence;

▫    Include clinical reasoning for the differential diagnoses during the oral case presentation;

▫    Review available resources in the department and describe the discharge rationale and plan for each patient.

At the end of the EM block, Resident X will:

▫    Be reevaluated by faculty using the PC-mSDOT either in the clinical setting or extra-clinical setting with an objective structured clinical exam (OSCE) or a simulation exercise.

Have shift evaluations been reviewed with the PD or another member of the residency leadership?

Resident Y has deficiencies with at least three PCMs (PC4, PC6, and PC7). The remediation plan for this resident may include:

During clinical shifts, Resident Y will be required to:

▫    Describe the reevaluation and discharge plan for all patients prior to discharge;

▫    “Check-in” with supervising faculty and residents at defined intervals during shifts to report the progress of therapeutic interventions and the ED course;

▫    Perform frequent rounds on patients to ensure that therapeutic interventions and care plans are executed in a timely fashion;

▫    Discuss clinical reasoning for differential diagnosis during case presentations;

▫    Describe discharge plans for patients, including acknowledgment of patient and/or family concerns, safety issues, financial, or compliance barriers; 

▫    Personally discharge 10 patients and review medications, follow-up information, and return precautions under direct faculty observation;

▫    Personally schedule follow-up appointments for a specific number of discharged patients.

Outside of the clinical setting, Resident Y will be required to:

▫    Attend biweekly simulation and oral board sessions that require a reassessment of interventions, disposition, and discharge planning. Designated faculty will supervise these sessions and immediate direct feedback will be given to the resident.

At the end of the remediation period, the resident will:

▫    Be reevaluated by faculty using the PC-mSDOT either in the clinical setting or extra-clinical setting with an OSCE or a simulation exercise;

▫    Have shift evaluations reviewed with the PD or another member of the residency leadership;

▫    Have in-training exam scores closely monitored to assess medical knowledge.

## Discussion

Resident remediation is prevalent in EM. In a survey of ACGME-accredited EM programs, Silverberg et al. found that 90% of program respondents had at least one resident on remediation within the previous three years [21]. The same study demonstrated that the prevalence of remediation in EM residencies is 4.4% with deficiencies in patient care being the second most common competency being remediated (46.6%) [21].

Among the challenges of remediation, PDs have difficulty with identifying residents in need of remediation, diagnosing the cause of their underlying deficiencies, and remediating them [22]. Residents failing to meet expectations may be identified in several ways, including the review of end-of-rotation evaluations, CCC meeting assessments, or a resident’s semi-annual review. However, waiting to uncover issues during infrequently scheduled evaluations may lead to a delay in the identification of deficiencies. The literature supports that post-rotation assessments completed by faculty are not helpful in identifying those residents who are struggling [23]. Moreover, several studies have shown that informal emails, telephone calls, and hallway/“curbside conversations” (rather than standardized assessments) are more common methods to raise concerns about resident competency [24-25]. Our proposed PC-mSDOT provides a resource for the early identification of residents who are not achieving appropriate milestone levels for their year of training. Moreover, the PC-mSDOT may be utilized to assess the progress of a resident who is undergoing remediation.

Studies have also demonstrated that resident remediation requires substantial resources [26-27]. Many PDs recognize the growing need for remediation toolkits, resources, and best practices. Katz et al. published a novel approach to remediation using actual resident cases presented to a multidisciplinary panel of current and former program directors. This panel utilized a four-step approach to create an expert consensus to develop a remediation plan of action [28]. However, with the development and implementation of milestones, the need for specific tools for the assessment and remediation of milestone deficiencies has arisen. There have been several remediation strategies that have been published in the recent literature. Williamson et al. published remediation strategies for systems-based practice (SBP) and practice-based learning and improvement (PBLI) milestones that may be applied across all specialties [29]. Similarly, Regan et al. published remediation methods for deficiencies in the interpersonal and communication skills (ICS) and professionalism milestones that may be utilized by all specialties [30]. The milestones that are focused on SBP, PBLI, ICS, and professionalism are more easily generalized across specialties than the PCMs. Although there is some commonality to various aspects of PC, such as history and physical examination skills, there are many more facets that are specialty-specific. There are currently no published tools or strategies for remediation of EM PCMs. It is the authors’ hope that the PC-mSDOT and the PCM remediation rubric offered by the CORD-RTF will be instrumental in assisting PDs in successful resident remediation.

The authors acknowledge that there are limitations to these remediation tools. First, the PC-mSDOT has yet to be validated. This is an ongoing process that clinician educators are currently working towards. Since there are currently no specific evidence-based “best practices” for remediation of PC, our toolkit was based on the expert consensus of the CORD RTF. Further review of the remediation outcomes will need to be tracked over time to establish best practices.

## Conclusions

EM program leadership can use the PCM-mSDOTs to identify resident strengths and areas for improvement, track resident progress, and initiate remediation plans. The PCM remediation rubric may a useful tool to formulate an individualized remediation plan for any resident with deficiencies at various milestone levels.
